# Aging and Viral Evolution Impair Immunity Against Dominant Pan‐Coronavirus‐Reactive T Cell Epitope

**DOI:** 10.1002/eji.202551888

**Published:** 2025-07-28

**Authors:** Lucie Loyal, Karsten Jürchott, Ulf Reimer, Lil Meyer‐Arndt, Larissa Henze, Norbert Mages, Jak Kostrzanowski, Bernhard Reus, Maike Mangold, Beate Kruse, Manuela Dingeldey, Birgit Sawitzki, Janine Michel, Marica Grossegesse, Karsten Schnatbaum, Holger Wenschuh, Andreas Nitsche, Nils Lachmann, Bernd Timmermann, Claudia Giesecke‐Thiel, Julian Braun, Florian Kern, Andreas Thiel

**Affiliations:** ^1^ Si‐M/“Der Simulierte Mensch” a science framework of Technische Universität Berlin and Charité ‐ Universitätsmedizin Berlin Berlin Germany; ^2^ Berlin Institute of Health (BIH) at Charité – Universitätsmedizin Berlin Immunomics ‐ Regenerative Immunology and Aging Berlin Germany; ^3^ JPT Peptide Technologies GmbH Berlin Germany; ^4^ NeuroCure Clinical Research Center Charité – Universitätsmedizin Berlin corporate member of Freie Universität Berlin Humboldt‐Universität zu Berlin and Berlin Institute of Health Berlin Germany; ^5^ Department of Neurology with Experimental Neurology Charité – Universitätsmedizin Berlin corporate member of Freie Universität Berlin Humboldt‐Universität zu Berlin and Berlin Institute of Health Berlin Germany; ^6^ Max Planck Institute for Molecular Genetics Berlin Germany; ^7^ Department of Informatics School of Engineering and Informatics University of Sussex Brighton UK; ^8^ Department of Clinical and Experimental Medicine Brighton and Sussex Medical School Brighton UK; ^9^ Translational Immunology Berlin Institute of Health (BIH) Berlin Germany; ^10^ Highly Pathogenic Viruses Centre for Biological Threats and Special Pathogens WHO Reference Laboratory for SARS‐CoV‐2 and WHO Collaborating Centre for Emerging Infections and Biological Threats Robert Koch Institute Berlin Germany; ^11^ Charité ‐ Universitätsmedizin Berlin Institute for Transfusion Medicine Tissue Typing Laboratory Berlin Germany; ^12^ Germany Berlin Institute of Health (BIH) at Charité – Universitätsmedizin Berlin Immunomics ‐ Regenerative Immunology and Aging Berlin Germany

**Keywords:** aging, cross reactivity, pan‐coronavirus, SARS‐CoV‐2, T cells

## Abstract

Immune evasion by escape mutations subverts immunity against SARS‐CoV‐2. A role of pan‐coronavirus immunity for more durable protection is being discussed, but has remained understudied. We here investigated the effects of age, mutations, and homo‐/heterologous vaccination regimens on the dominant pan‐coronavirus‐specific cellular and humoral epitope iCope after SARS‐CoV‐2 infection and vaccination in detail. In older individuals, the quantitatively and qualitatively reduced iCope‐reactive CD4^+^ T cell responses with narrow TCR repertoires could not be enhanced by vaccination and were further compromised by emerging spike mutations. In contrast, pan‐coronavirus‐reactive humoral immunity was affected only by mutations and not by age. Our results reveal a distinct deficiency of the dichotomous layer of pan‐coronavirus immunity in the older, critical for long‐term protection against SARS‐CoV‐2 variants.

## Graphical abstract

The immunodominant pan‐coronavirus epitope iCope spans amino acids 816–830 within the fusion domain of the SARS‐CoV‐2 spike glycoprotein. This region exhibits a comparatively low mutation rate, especially when contrasted with the receptor‐binding domain (RBD). Antigen‐specific T cell responses against iCope are detectable even in unexposed individuals, are modestly enhanced following SARS‐CoV‐2 infection, and are robustly boosted by two doses of the BNT162b2 vaccine (BioNTech/Pfizer). Strikingly, in individuals over 70 years of age, vaccination fails to enhance the quantity and quality (avidity) of the iCope‐specific T cell response, likely due to a restricted TCR repertoire targeting this epitope. In contrast, iCope‐specific antibody titers do not exhibit an age‐dependent decline.

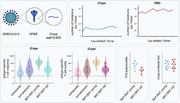

## 1 Introduction

The severity of coronavirus disease 2019 (COVID‐19) caused by severe acute respiratory syndrome coronavirus 2 (SARS‐CoV‐2) depends on a variety of factors, including socioeconomic status, genetics, comorbidities, medication (immunosuppressants), pre‐existing cross‐reactive immunity, and, in particular, age [1, 2, 3, 4, 5]. The rate of severe complications requiring hospitalization is low in the younger, but increases beginning at the age of 30, resulting in a high fatality rate among the >80‐year‐olds [6]. First‐generation vaccines provided effective protection against the Wuhan‐WT SARS‐CoV‐2; however, the generally limited vaccine effectiveness in the older population remains a concern [7, 8, 9]. In addition, SARS‐CoV‐2 is undergoing rapid viral evolution, generating variants of concern (VOC) characterized by increased transmissibility, altered disease severity, and evasion of neutralization by antibodies induced during previous vaccination and infection [10, 11, 12]. Moreover, in general, a rapid decline in mucosal antibodies providing direct protection at viral entry sites has been observed [13]. These observations have dashed the hope that SARS‐CoV‐2 vaccines would be able to induce long‐term, sterile humoral immunity. It is now rather emphasized that specific memory T cells provide a main layer for long‐term protection since they can react even against highly mutated variants due to their broad epitope coverage [14, 15, 16, 17], including dominant pan‐coronavirus epitopes [18, 19, 20]. Such pan‐coronavirus‐reactive T cells lead to rapid cellular and humoral responses in the early phase of SARS‐CoV‐2 infection, which positively correlate with faster and better T cell and antibody responses after infection and vaccination [4, 5, 8, 18, 21, 22, 23, 24]. This observation links the presence of pre‐existing pan‐coronavirus‐specific T cells directly to the success of early antibody induction. However, with age, spike‐specific pan‐coronavirus cellular immunity declines in older individuals [8, 18, 25, 26]. Whether this apparently critical component of cellular immunity can be restored in the older and how virus evolution affects cellular immunity directed at conserved spike protein regions is unknown. Importantly, cellular and humoral pan‐coronavirus reactivity is dominated by an immunodominant coronavirus peptide (iCope) sequence located within the fusion peptide domain of the spike protein (amino acids 816–830) [18, 19, 27, 28, 29, 30, 31, 32]. We comprehensively investigated the extent to which existing and potential future spike mutations impact iCope‐specific cellular and humoral responses in unexposed individuals of different ages upon infection and vaccination.

## 2 Results

### 2.1 The iCope Sequence Is Conserved in SARS‐CoV‐2 Variants

**FIGURE 1:**
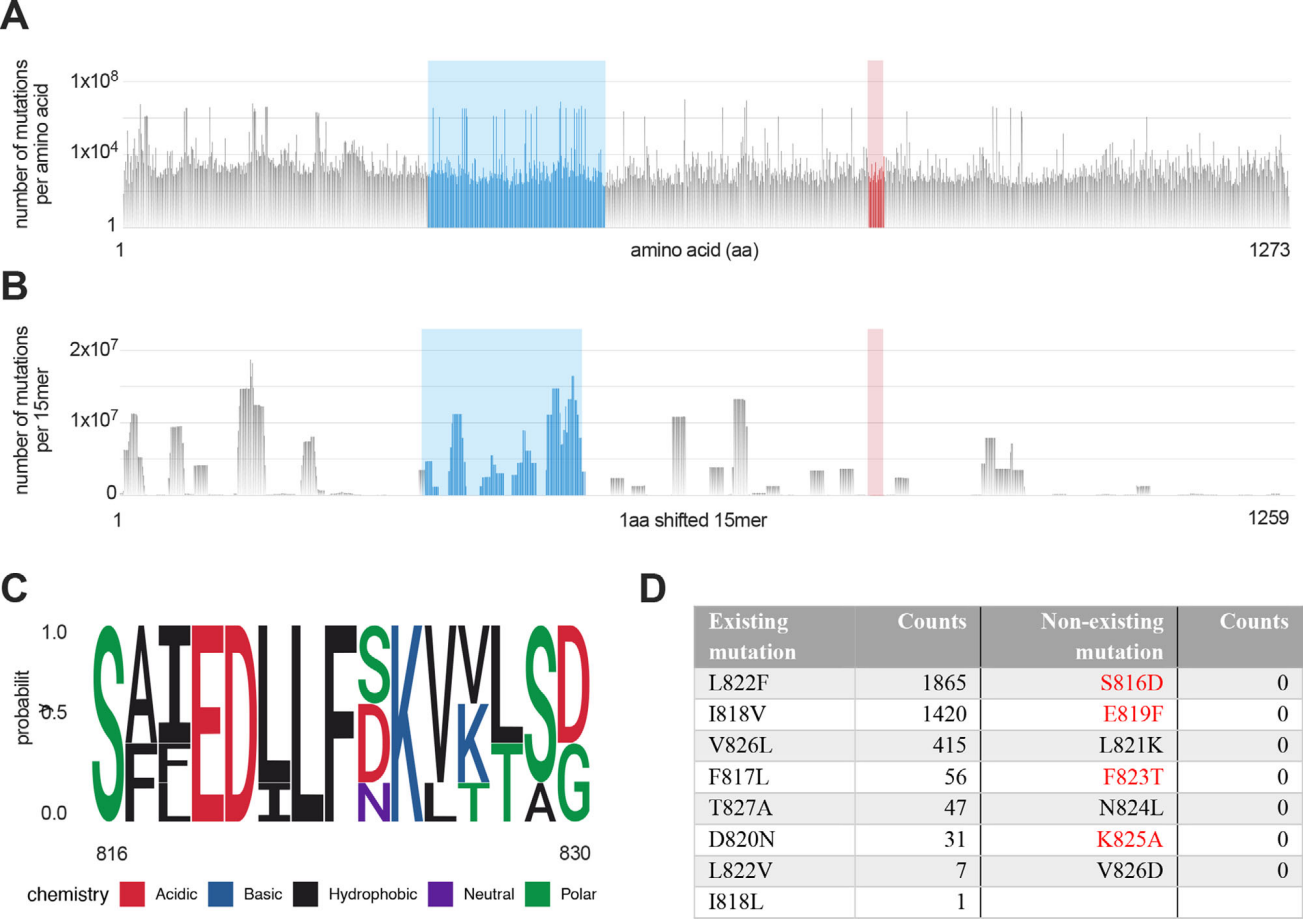
iCope mutation rate and sequence homology. (A) Mutations in 10.8 mio spike sequences per amino acid (GISAID, May 15, 2022). Amino acids 816–830 (highlighted in red) compared with other amino acids within the spike, including RBD binding domain covering aa 333–526 (highlighted in blue). (B) Numbers of 1aa shifting 15mers carrying a mutation throughout spike (GISAID, May 15, 2022). 15mers covering the iCope sequence are indicated in red. (C) Motif analysis of amino acid conservation of SARS‐CoV‐2 S816‐830 in endemic coronaviruses (229E, NL63, OC43, HKU1). (D) Number of sequences with indicated mutations that were reported in GISAID by September 2021 and additionally generated nonexisting mutations. Those replacing the most conserved amino acids are highlighted in red.

First, we aimed to understand if and to what extent the SARS‐CoV‐2 spike S816‐830 sequence SFIEDLLFNKVTLAD (iCope) is conserved. Therefore, we assessed the mutation rate of iCope in comparison to other sequences within the spike. Within 10.8 mio globally reported spike sequence reads with unique mutations (GISAID, May 2022), the number of mutations per amino acid was comparatively low in iCope (Figure 1A). Similarly, the summarized mutation rates in a scan of 15mers with 1 amino acid shifting revealed that iCope is a very inert sequence, especially compared with the highly mutated RBD sequence (aa 333–526) (Figure 1B). In line with this, none of the existing variants of concern (VOC) from alpha to omicron, including the omicron subvariants BA1‐5, showed mutations within iCope. A comparison of iCope with the respective sequence of endemic coronaviruses (NL63, 229E, OC43, HKU1) demonstrated high conservation of the amino acids S816, E819, D820, L822, F823, and K825 (Figure 1C). Only four out of 15 positions (N824, T827, L828, A829) displayed variation in the amino acid groups' chemical properties, while at positions F817, I818, and L821, conservative substitutions retain the hydrophobic characteristics of the amino acids. Of 2.3 mio reads in the GISAD database by September 2021, eight mutations affecting iCope with different incidences were reported (Figure 1D). Consequently, to investigate potential alterations in T cell reactivity, we generated peptides for the identified mutations, including the frequent hydrophobic‐to‐hydrophobic mutations: L822F, I818V, F817L, and V826L. While the first three mentioned mutations are novel mutations, that is, only occurring in SARS‐CoV‐2, the V826L mutation is shared by several HCoVs. The mutation T827A exchanges the polar T for the nonpolar A, whereas T827V, where T is exchanged for the equally hydrophobic V, is also found in other HCoVs. D820N generates a novel mutation affecting an otherwise highly conserved amino acid. In order to also assess the effects of some drastic hypothetical changes in the amino acid sequence including changes to most conserved regions S816, E819, L821, F823, and K825 we additionally generated peptides with the following hypothetical mutations: S816D: polar to acidic, E819F: acidic to hydrophobic, L821K hydrophobic to basic, F823T: hydrophobic to polar and K825A: basic to nonpolar, N824L: polar to hydrophobic and V826D: hydrophobic to acidic (Figure 1D).

### 2.2 The Amino Acids S819‐826 Are Critical for iCope‐Specific T Cell Responses

**FIGURE 2:**
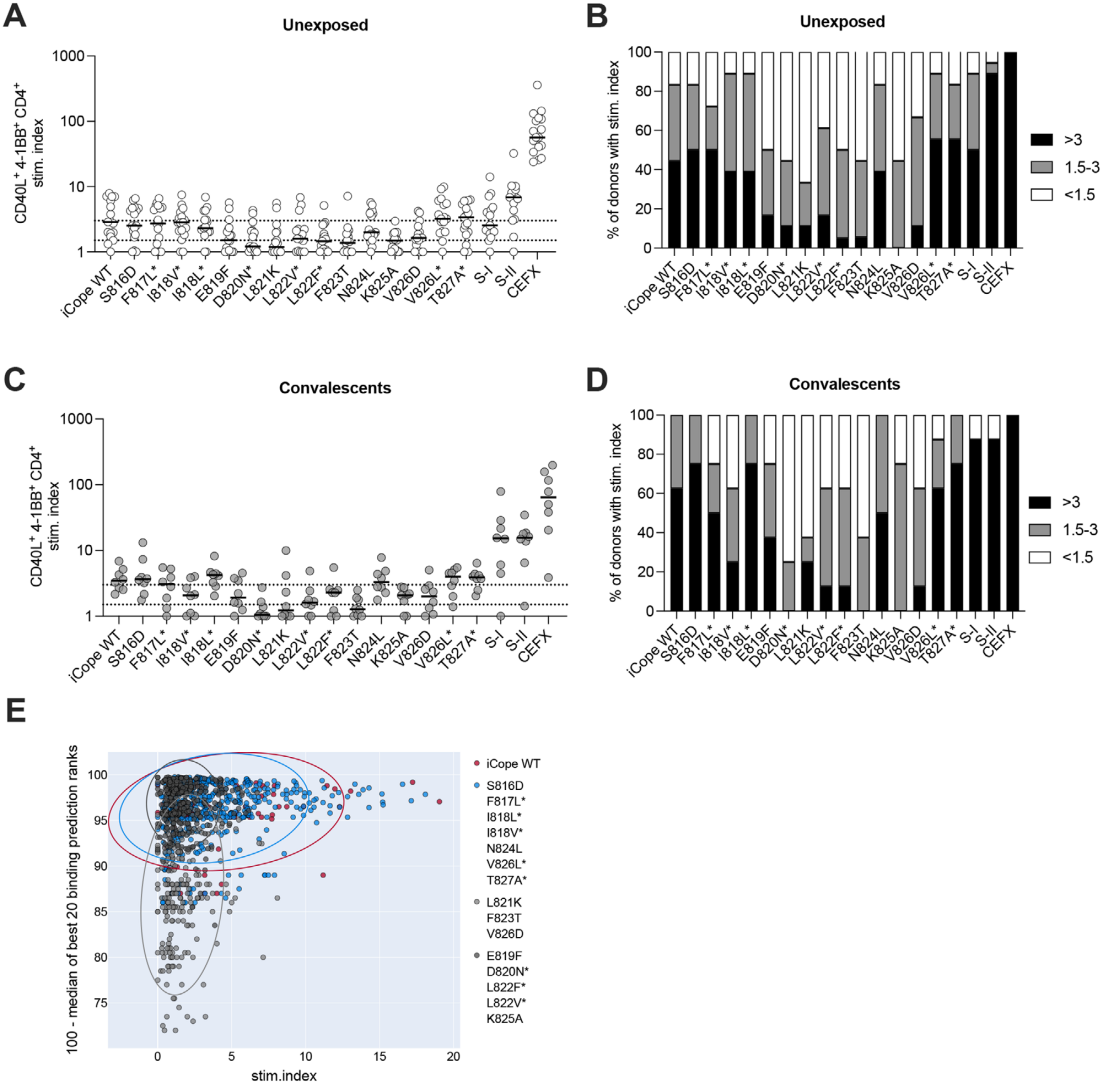
Mutations in S819‐826 impair iCope‐specific T cell responsiveness in unexposed and convalescents. Ex vivo stimulation of PBMCs from unexposed individuals (*n* = 17) and COVID‐19 convalescents (*n* = 8) with iCope WT or different mutated iCope peptides and control pools S‐I and S‐II, and CEFX as positive control. (A, C) The percentage of CD40L^+^4‐1BB^+^ CD4^+^ T cells among stimulated PBMC was divided by the percentage of these cells among unstimulated PBMC to determine the stimulation index (stim. index) shown on the y‐axis. Bars show the median. Dotted lines indicate a stim. index of 1.5 and 3. All values below 0.1 were set at 0.1 for display. (B, D) Bar plots show the proportions of individuals with the indicated peptide or peptide pool stimulations with a stim. index >3 (black), 1.5‐3 (grey) or <1.5 (white). (E) MHCII peptide binding was predicted for all combinations of the iCope/mutations peptides for all 96 donors from Table S1 using IEDB. The median of the best 20 prediction ranks was calculated for each allele and plotted against the stimulation index from the stimulations with iCope or its mutant peptides. Color codes indicate iCope wildtype peptide (red), mutated iCope peptides with no to weak negative effect on T cell activation (blue), and mutated peptides with strong negative effect on T cell activation (grey). Those with IEDB‐based, predicted negative effects are separated from the others by light grey dots. * indicates documented iCope mutations.

We examined the effects of described and potential amino acid mutations on iCope‐specific CD4^+^ T cell reactivity by comparing peptide stimulations of wildtype (WT) iCope to the different mutated iCope peptides as well as N‐terminal (S‐I, aa 1–643) and C‐terminal (S‐II, aa 633–1273) spike peptide pools. In 44% of young, unvaccinated, and uninfected donors (unexposed, Table S1), we detected a strong response to stimulation with WT‐iCope (Figure 2A,B), and 9 out of 15 assessed iCope mutations affected T cell responsiveness. Despite the high conservation of S816 across coronaviruses, the substitution of the polar S by the acidic D had no effect on T cell reactivity. While the V826D substitution impaired T cell activation, the conserved V826L mutation did not. The widely prevalent I818V mutation also had no effect on T cell reactivity. In contrast, the F817L mutation caused a decrease in four donors, indicating a strong HLA‐dependent effect. The strongest decrease of T cell reactivity was observed for mutations within the region S819‐826 (Figure 2B,D), and thus, except for aa 824, the S819‐826 region appears highly critical for effective T cell activation. Interestingly, recovery from COVID‐19 does not appear to substantially increase the abundance of iCope‐reactive CD4^+^ T cells (Figure 2C), yet these cells have been shown to be engaged early during infection and vaccination with beneficial effects [18]. In convalescent donors, the effects of the different mutations follow the same pattern as in unexposed donors (Figure 2D). Analysis of MHC class II /peptide binding prediction (IEDB.org) in combination with iCope‐specific T cell responses comprising all 96 donors of Table S1 indicated that the mutations S816D, F817L, I818L, I818V, N824L, V826L, T827A did not significantly affect peptide binding and T cell responsiveness (Figure 2E; Figure S1). While L821K, F823T, and V826D were identified in silico as poor MHC binders (depicted in light grey, Figure 2E) also the mutations E819F, D820N, L822F, L822V, and K825A impair the anti‐iCope response (depicted in dark grey, Figure 2E). Altogether, this identifies the S819‐826 region as critical for iCope‐specific T‐cell responses.

### 2.3 Vaccination Boosts iCope Immunity in the Younger but Not in the Older

**FIGURE 3:**
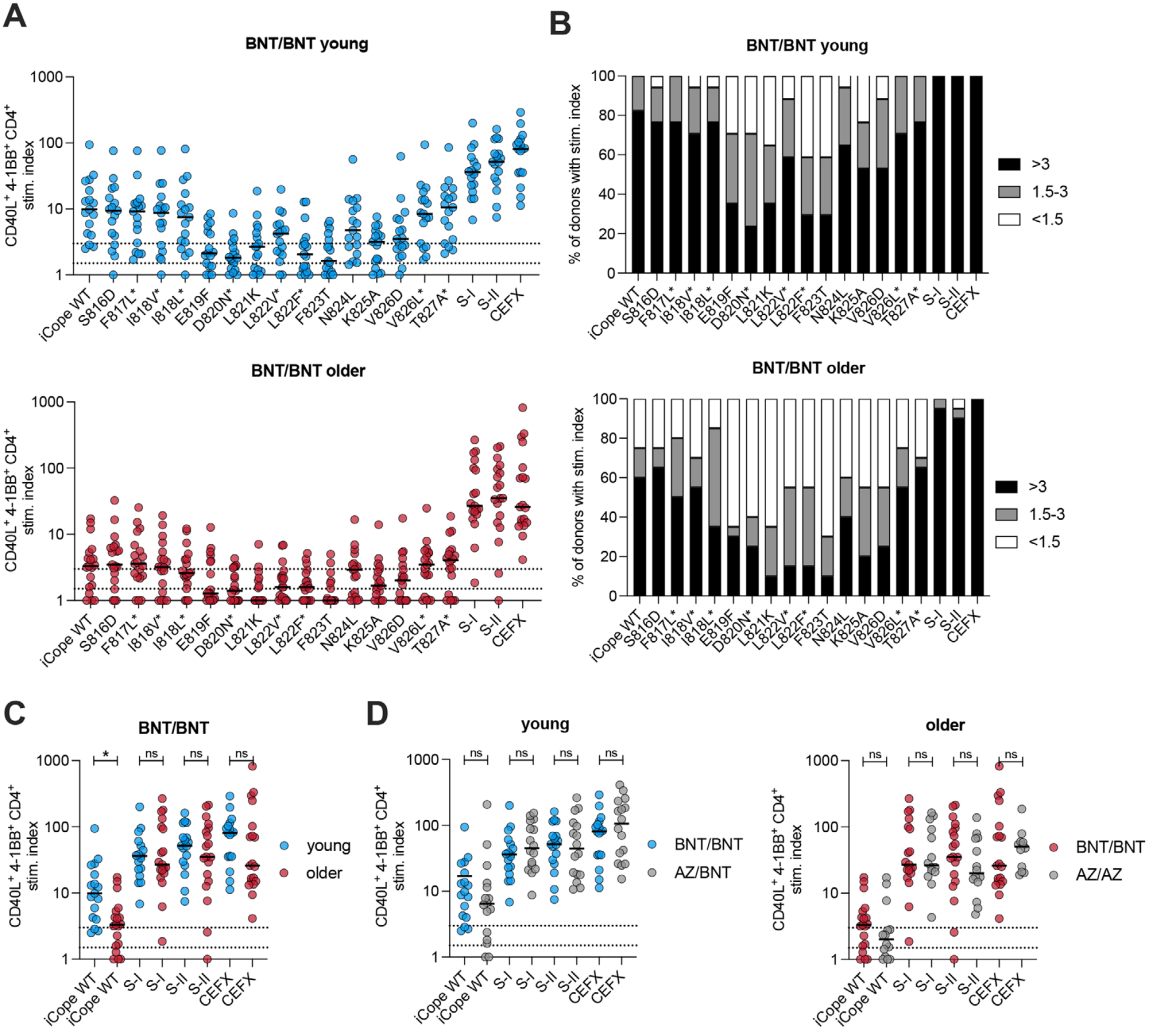
iCope responsiveness in vaccinated donors is age dependent. Ex vivo stimulation of PBMCs from young or older BNT/BNT (*n* = 18/19) vaccinated individuals with iCope WT or different mutated iCope peptides and control pools S‐I, S‐II, and CEFX. * indicates documented iCope mutations. (A, C, D) The percentage of CD40L^+^4‐1BB^+^ CD4^+^ T cells among stimulated PBMC was divided by the percentage of these cells among unstimulated PBMC to determine the stimulation index (stim. index) shown on the y‐axis. Bars show the median. Dotted lines indicate a stim. index of 1.5 and 3. All values below 0.1 were set at 0.1 for display. (B) Bar plots show the proportions of individuals with the indicated peptide or peptide pool stimulations with a stim. index >3, 1.5‐3 or <1.5. (C) Comparison of young and older BNT/BNT vaccinated donors under the indicated stimulation conditions. (D) Comparison of young BNT/BNT with young AZ/BNT vaccinated donors and older BNT/BNT with older AZ/AZ vaccinated donors under the indicated stimulation conditions. *p < 0.05, **p < 0.01, ***p < 0.001 and ns for *p *< 0.05 (Student's *t*‐test). BNT: BNT162b2 mRNA COVID‐19 vaccine (BioNTech); AZ: ChAdOx1 COVID‐19 vaccine (AstraZeneca).

We have previously shown that vaccination with the BNT162b2 (BNT, BioNTech/Pfizer) mRNA COVID‐19 vaccine leads to activation of iCope‐specific T cells with beneficial effects. However, the level of pre‐existing S‐II spike peptide pool‐specific cross‐reactive T cells, dominated by iCope‐specific cells, decreased with age [18]. To address the consequences of this on vaccine immunogenicity in the older, we compared the CD4^+^ T cell responses to iCope upon homologous BNT162b2 vaccination in young (age <40, mean 30.8 years) and older individuals (age >60, mean 76.5 years, Table S1). While the impact of mutations remained comparable in both groups, the total amount of responsive T cells in the older, triple‐vaccinated donors decreased to a level similar to that of young, unexposed donors (Figure 3A,B, cf. Figure 2A). When we compared the effects of the different vaccination regimens, we found that T cell responsiveness was not affected by vaccine combination, but again by age (Figure 3C,D; Figure S2A). While vaccination enhanced iCope‐specific cellular responses in the younger and also in potential mutants (cf. Figures 2A and 3A), neither homologous nor heterologous vaccination could rescue the low iCope‐specific CD4^+^ T cell responses in older individuals (Figure 3D).

### 2.4 Age Affects the Quality of the iCope Specific T Cell but Not B Cell Response after Infection and Vaccination

**FIGURE 4:**
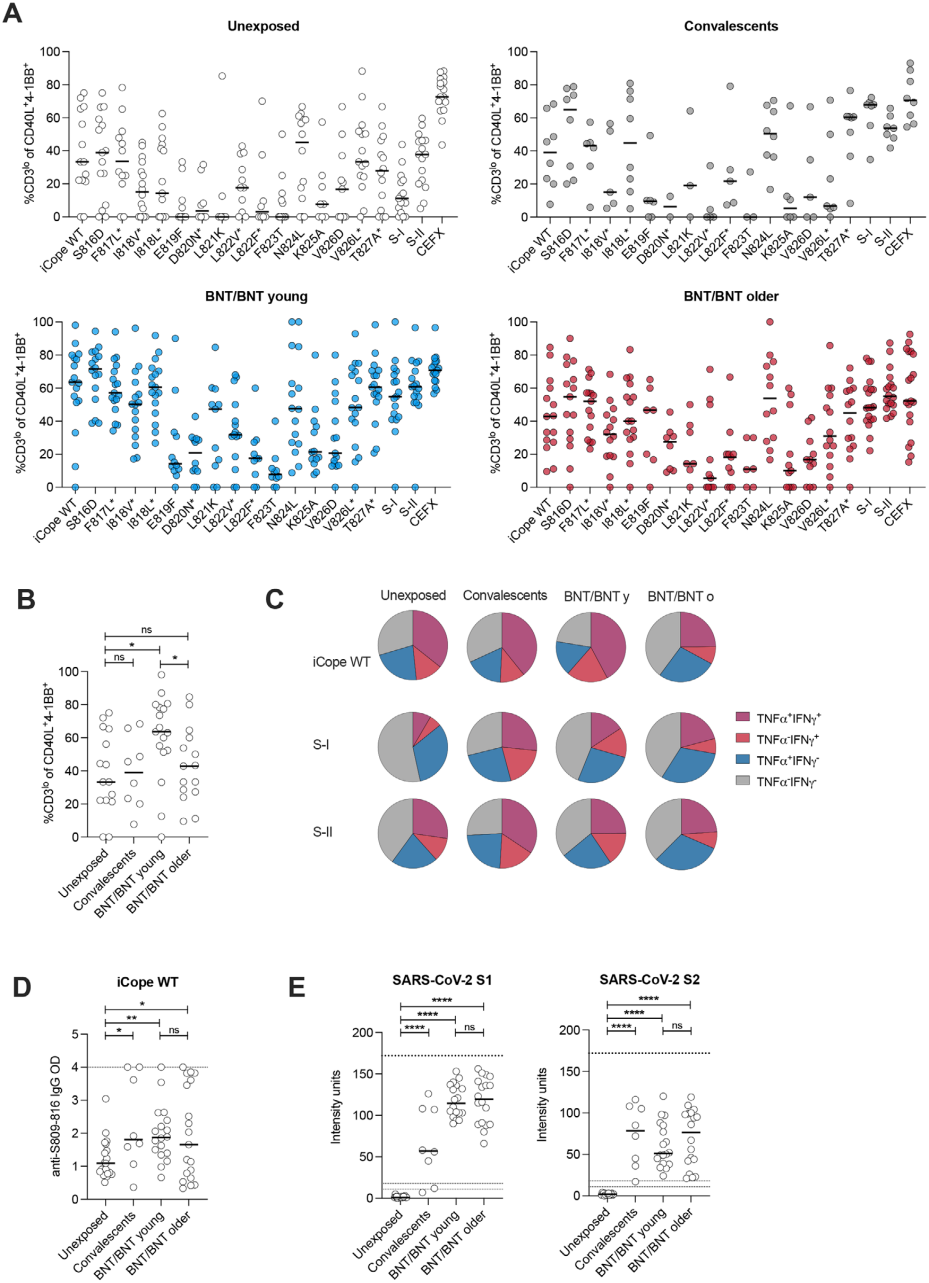
The quality of iCope responsiveness is impaired in older individuals. Ex vivo stimulation of PBMCs from unexposed (*n* = 17), convalescent (*n* = 8), young BNT/BNT vaccinated (*n* = 18), and older BNT/BNT vaccinated (*n* = 19) individuals with iCope WT or different mutated iCope peptides and the control pools S‐I, S‐II, and CEFX. * indicates documented iCope mutations. (A, B) Frequencies of CD3^lo^ cells in CD40L^+^4‐1BB^+^ CD4^+^ T cells. CD3^lo^ frequencies are shown for T cell responses with a stim. index ≥1.5. Bars show the median. (C) Proportion of IFN‐γ and/or TNF‐α producing T cells among CD40L^+^4‐1BB^+^ CD4^+^ T cells in the indicated cohorts after stimulation with iCope WT, S‐I, or S‐II. (D) Optical density (OD) of anti‐S809‐826 wildtype (WT) peptide IgG (ELISA) in unexposed (*n* = 17), convalescents (*n* = 8), BNT/BNT young (*n* = 18), and older BNT/BNT (*n* = 19). Bars show the median. (E) Levels of anti‐S1‐ or anti‐S2‐IgG binding antibody intensity units in indicated cohorts. Dotted lines indicate lower cut‐off (set at 11) for definitive negative values and a range from 11–18 for values classified as positive with uncertainty. Bars show the median. *p < 0.05, **p < 0.01, ***p < 0.001, ****p < 0.0001, ns for *p *< 0.05 (Student's *t*‐test).

Next, we assessed whether mutations in the iCope sequence and/or aging affect the quality of the iCope‐specific T cell responses, that is, TCR avidity and effector functions, upon infection or vaccination. We used the CD3 surface expression level among antigen‐reactive CD4^+^ T cells as readout as the degree of CD3 downregulation (CD3^lo^) on in vitro activated T cells can serve as a surrogate for TCR avidity [16]. The TCR avidity of the iCope‐specific T cells was significantly reduced by mutations within the aa 819–823 region of spike as well as in position aa 825–826 in all examined cohorts (Figure 4A; Figure S3A). In general, peptides with mutations leading to reduced MHC‐binding also induced lesser CD3 downregulation; however, there were some exceptions, such as F817L and I818L, with a negative impact on the TCR avidity but not overall T cell responsiveness (Figure 4A; Figure S3A). The L822V replacement reduced the number of reactive cells, but the few responding cells showed high avidity for the target. In the course of SARS‐CoV‐2 infection, high‐avidity T cells targeting S‐I and S‐II peptides were generated, but no substantial changes in the avidity of iCope‐specific T cells were observed after clearance of the infection. In contrast to this, the homologous BNT prime‐boost (BNT/BNT) resulted in the expansion of high‐avidity iCope‐specific T cell clones in the young, but again not in the older individuals (Figure 4A,B). Moreover, remaining iCope‐specific T cells in vaccinated older people displayed a reduced capacity for TNF‐α and IFN‐γ secretion (Figure 4C), two cytokines fundamental in the host's combat against SARS‐CoV‐2 infection. Mutations, too, led to an impaired polyfunctional response in the remaining iCope‐specific T cells (Figure S3B). In contrast, anti‐iCope antibodies were increased upon infection and vaccination along with the SARS‐CoV‐2 S1‐ and S2‐specific humoral immunity, with no detectable difference between the young and the older vaccinees (Figure 4D,E). When assessing the impact of different mutations on antibody binding, F823T together with S816D, I818V, and E819F displayed the strongest negative effect on antibody binding, whereas, for T cells, any tested mutation affecting the region between E819F‐V826D had a strong negative effect (Figure S4A). In summary, aging drastically affects the composition and functional capacity of iCope‐specific T cells but not humoral responses. Mutations, however, seem to affect both cellular and humoral immunity, albeit to different extents.

### 2.5 Reduced iCope T Cell Responsiveness in the Older Is Associated with a Thinned‐out TCR Repertoire

**FIGURE 5:**
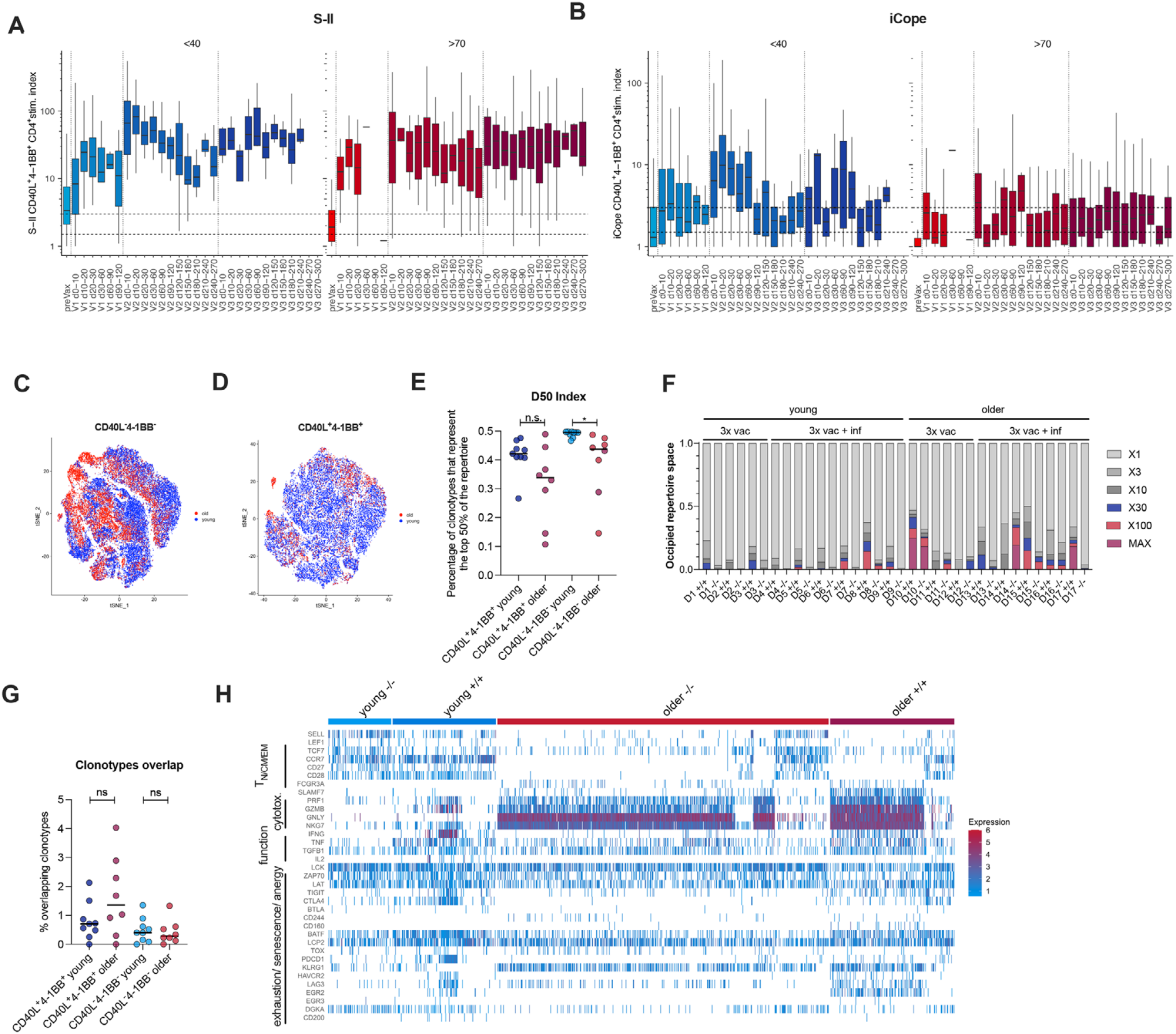
Older people possess functional iCope‐reactive T cells but with limited clonal breadth. (A, B) Ex vivo stimulation of PBMCs from young (<40 years, blue, *n* = 507) or older (>70 years, red, *n* = 1267) individuals with S‐II (A) or iCope (B) prior to first vaccination (preVax) or at indicated times after the first, second, or third dose of vaccination (V1‐3). The percentage of CD40L^+^4‐1BB^+^ CD4^+^ T cells among stimulated PBMC was divided by the percentage of these cells among unstimulated PBMC to determine the stimulation index (stim. index) shown on the y‐axis. (C, D) Single‐cell gene expression of FACS‐purified iCope reactive CD40L^+^4‐1BB^+^ and CD40L^−^4‐1BB^−^ CD4^+^ T cells of *n* = 9 young (<43 years) and *n* = 8 older (>76 years) donors. tSNE visualization of CD40L^+^4‐1BB^+^ (C) and CD40L^−^4‐1BB^−^ (D) CD4^+^ T cell clustering of young (blue dots) and older (red dots) donors. (E) D50 diversity index indicating the number of clonotypes occupying the top 50% of the TCR repertoire. (F) Proportion of clonotypes with specific counts in indicated samples of the different donors (D1‐6). D1‐3 are the triple vaccinated young donors without, D4‐9 with recent infection, D10‐12 triple vaccinated older donors without, 13–17 with recent infection. (G) Frequencies of overlapping clonotypes between activated CD40L^+^4‐1BB^+^ and inactivated CD40L^−^4‐1BB^−^ CD4^+^ T cells in young and older donors. Bars show the median. (H) Heatmap of gene expression signatures of curated gene sets (as given in the Material and Methods section) for naïve and effector T cell characteristics as well as cytotoxicity, anergy, exhaustion and senescence in overlapping clonotypes between activated CD40L^+^4‐1BB^+^ (+/+) and inactivated CD40L^−^4‐1BB^−^ (−/−) CD4^+^ T cells. *p < 0.05, **p < 0.01, ***p < 0.001, ****p < 0.0001, ns for *p *< 0.05 (Student's *t* test).

To delineate the age‐associated quantitative and qualitative differences of iCope‐specific T cells in more detail and determine whether additional vaccinations could rescue the observed scrambled responses in the older, we assessed iCope and S‐II responses in individuals aged <40 or >70 who had three vaccinations but had no indicators of infection (anti‐nucleocapsid IgG titer and/or antinucleocapsid T cell response) (Figure 5A,B; Figure S5A,B). We observed robust (>200 days) T cell frequencies against S‐II in the older (Figure 5A; Figure S4A); however, the number of donors that were weak‐to‐nonresponsive against the pan‐coronavirus‐specific iCope remained high even after the third vaccine dose (Figure 4B; Figure S5B). To deconvolve the reason for the altered iCope‐specific T cell responses in the older we conducted single‐cell RNA‐sequencing of FACS‐purified iCope‐reactive CD4^+^ T cells (CD40L^+^4‐1BB^+^) and as a control iCope‐nonreactive (CD40L^−^4‐1BB^−^) CD4^+^ T cells from 3 young (<30 years) and 3 older (>80 years) donors 3 months after the third dose of BNT162b2 vaccine and 6 young and 5 older equally vaccinated donors with SARS‐CoV‐2 infection in the last 3 months prior sampling (Table S2). iCope‐nonreactive CD4^+^ T cells showed an age‐related shift on the molecular level, whereas iCope‐specific T cells showed no age‐related cell‐intrinsic gene expression differences, except for a distinct population of cytotoxic CD4^+^ T cells, the red cluster in the upper left (Figure 5C,D). However, the diversity of the total and iCope‐responsive TCR repertoire was reduced in the older ones and characterized by an enrichment of only a few clones (Figure 5E,F). Assessing the proportion of iCope‐specific TCR CDR3 sequences of CD40L^+^4‐1BB^+^ within CD40L^−^4‐1BB^−^ T cells, we observed no differences between young and older donors, arguing against an age‐dependent activation deficiency (Figure 5G). Nonactivated iCope‐specific clones among CD40L^−^4‐1BB^−^ T cells in older mice also did not display any sign of exhaustion, anergy, or senescence but a prominent cytotoxic CD4^+^ T cell signature (Figure 5H). Our data suggest that the reduction in quantity and quality of iCope‐specific T cells in older individuals is caused by a narrowing of the TCR repertoire, accompanied by a loss of clones with high TCR avidity and effector functions and differentiation into cytotoxic CD4^+^ T cells.

## 3 Discussion

SARS‐CoV‐2 immunity varies due to pre‐existing reactivities, vaccinations, encountered infections, and individual susceptibility to severe disease, which is influenced by age, comorbidities, medications (e.g., immunosuppression), and genetics. Newly arising SARS‐CoV‐2 variants, as well as potential future coronavirus spillovers, can differ drastically with respect to their immunogenicity and hence the immune system's ability to recognize and neutralize them at sites of viral entry.

Due to the MHC polymorphism, mutations may not only result in the loss of selected individual T cell epitopes but also generate new ones [33]. However, not all combinations of vaccinations and infections with SARS‐CoV‐2 variants boost T‐cell responses. Hybrid immunity resulting from infection with Wuhan‐WT SARS‐CoV‐2 followed by an Omicron infection results in “hybrid immunity dampening” with reduced T cell responses against the S1‐subunit [33]. Importantly, the effect is not observable when assessing S2‐subunit‐specific T cell responses, suggesting the largely unmutated S2 epitopes of spike can compensate for this effect.

The second most fundamental challenge for efficient long‐lasting immunity against SARS‐CoV‐2 and its emerging variants is the aging of the human immune system, which is associated with a loss of a broad polyclonal repertoire of naïve T cells caused by thymic involution beginning with puberty [34, 35, 36, 37]. Reduced thymic output has been demonstrated to affect primary vaccination in the older, even with live vaccines such as the yellow fever vaccine [38, 39]. Low numbers of naïve T cells and lower TCR diversity against SARS‐CoV‐2 epitopes were also associated with severe COVID‐19 [40, 41], and lower frequencies of IFN‐γ‐ and IL‐2‐producing T cells in SARS‐CoV‐2 have been reported in vaccinated older individuals [42]. Accordingly, pre‐existing pan‐coronavirus‐specific memory T cells may be a critical source of cellular immunity to enable timely reactivation of pre‐existing memory B cells, but also for the fast recruitment of new naïve B cell specificities [43, 44]. Pre‐existing cross‐reactive S2‐specific antibodies can prevent infection by blocking the fusion machinery of SARS‐CoV‐2, and IgA‐producing B cells have been demonstrated already after the first dose of vaccination [27, 45, 46, 47, 48, 49, 50, 51, 52]. Given the possible importance of neutralizing S2‐specific antibodies for controlling SARS‐CoV‐2 variants characterized by altered RBD regions, the evolution of these responses should be analyzed in more detail. Additionally, the mechanisms by which these responses are maintained in the older should be explored. It is of interest in this context that in influenza infection, rapid cellular responses mediated by pre‐existing cross‐reactive T cells are associated with protection from symptomatic infection and severe disease even in seronegative individuals [53, 54, 55]. In line with this, pre‐existing cross‐reactive T cells are able to provide T cell help in the early phase of infection or after vaccination and protect against SARS‐CoV‐2 infection [4, 18, 24]. Additionally, pre‐existing cross‐reactive T cells targeting the SARS‐CoV‐2 polymerase can abort infection and so contribute to the containment of virus spreading [5].

The pan‐coronavirus‐specific fusion peptide region in the spike contains antigenic determinants recognized by B cells and T cells that so far have remained relatively unaffected by novel mutations of SARS‐CoV‐2. This region may, therefore, be considered a natural target for the development of pan‐coronavirus‐specific vaccines. However, pre‐existing pan‐coronavirus‐specific cellular immunity declines with age, and we reveal here that the resulting deficiencies cannot be restored by homologous or heterologous vaccination in older individuals. While in young individuals a broad and diversified iCope‐specific clonotype repertoire is induced by vaccinations or in combination with infections, older people are not capable of such repertoire broadening. Our results suggest that the impaired pan‐coronavirus‐specific T‐cell responsiveness to SARS‐CoV‐2 vaccination is caused by a gradual loss of “best‐fit” clones, an effect also known from influenza [56, 57]. This is also in line with the recent observation that cross‐reactive responses are mainly established in early childhood and decline afterward [26]. Additionally, the remaining responses in the older appear to be characterized by a low TCR avidity toward iCope, which can result in the observed reduced polyfunctionality. Besides, we did not observe signatures of senescence, exhaustion, or anergy in iCope‐specific clones [58]. Even after a third vaccination, the lack of reactive clones in the repertoire rather than the unresponsiveness of existing clones (e.g., due to anergy or exhaustion) is the reason for impaired T cell responsiveness in the older. It has been emphasized that in many older individuals, a third dose of vaccine is necessary to boost an incomplete response against the complete spike protein achieved with two vaccinations [7]. However, we reveal here that even a booster vaccination may result in, albeit quantitatively equal, qualitatively still impaired responses.

Single‐cell RNA sequencing revealed an expanded iCope‐specific cytotoxic CD4^+^ T cell population in the older donors, which was partly not identified as antigen‐specific due to the absent CD40L expression in terminally differentiated cytotoxic CD4^+^ T cells [59]. Accumulation of cytotoxic CD4^+^ T cells is a general feature of the very old, but their enrichment has also been observed in adults compared with children and hospitalized patients compared with nonhospitalized individuals [60, 61]. Strong cytotoxic CD4^+^ T cell responses early in the infection were linked to defects in B cell responses, implying iCope‐ and therefore pan‐coronavirus‐specific T cells with cytotoxic properties might be a risk factor for severe disease [62].

The development of next‐generation pan‐coronavirus‐targeting vaccines focusing on intranasal administration could compensate for the observed alterations in the older if sufficient reservoirs of tissue‐resident T cells are still present that can be reactivated by the intranasal challenge. It has already been demonstrated that high titers of neutralizing antibodies can be induced in the nasally administered ChAdOx1 in hamsters [63] and phase I clinical trials with inactivated NDV‐HXP‐S vaccines [64]. Other first results are discouraging, revealing rather low induction of specific immunity [65]. Utilizing dominant anti‐SARS‐CoV‐2 epitopes derived from different SARS‐CoV‐2 proteins, a strong T‐cell induction was observed; however, the stability of these responses remains to be assessed [66]. Previous and recurrent encounters with endemic coronaviruses and, more recently, with different SARS‐CoV‐2 variants result in high levels of pan‐coronavirus reactive T‐ and B‐cells in the respiratory tract, as shown recently for bronchoalveolar lavage (BAL) and oropharyngeal lymphoid tissue [67, 68]. We propose that only with the efficient local (re‐)activation of these pan‐coronavirus‐reactive immune cells, long‐term immunity against SARS‐CoV‐2 and its emerging variants is achievable. In particular, in the elderly, this reservoir might be the only source of sufficiently broadly targeting immune cells against SARS‐CoV‐2, since the availability of broadly specific naïve T cells in the elderly is irrevocably decreased.

## 4 Materials and Methods

### 4.1 Study Participants

The age, gender, and comorbidities of all donors were recorded (Tables S1 and S3). Participants who had tested positive for SARS‐CoV‐2 RNA (RT‐qPCR from nasopharyngeal swabs) and displayed SARS‐CoV‐2‐related symptoms were classified as convalescent donors. All convalescent donors had mild symptoms (WHO severity grade < 3), and no hospitalization was required. The day of infection was set as day 3 before reported symptom onset. The vaccinated cohorts had the following intervals between their first and second dose of vaccine: homologous BNT162b2 vaccination: 3 weeks, homologous ChAdOx1 vaccination: 12 weeks, and heterologous ChAdOx1/BNT162b2 vaccination: 12 weeks. All enrolled participants were screened for SARS‐CoV‐2 subunit 1 (S1) and nucleocapsid (N) antibody titers. Two unexposed, two young BNT/BNT vaccinated, and one older BNT/BNT vaccinated individual were excluded from the analysis due to detectable S1 and/or N (unexposed) or N (vaccinated) IgG titers.

### 4.2 Coronavirus RT‐qPCR

RNA was extracted from 140 µL of wet nasopharyngeal swabs (Copan mini UTM) using the QIAamp Viral RNA Mini Kit and QIAcube Connect with the manual lysis protocol. SARS‐CoV‐2 RNA detection was performed using a simultaneous two‐duplex one‐step real‐time RT‐PCR assay with custom primers and probes (Metabion and Thermo Fisher Scientific) for SARS‐CoV‐2 E Gene and SARS‐CoV‐2 ORF1ab according to the RKI/ZBS1 SARS‐CoV‐2 protocol as described before [69]. Each one is duplexed with a control that either indicates potential PCR inhibition or proves the successful extraction of nucleic acid from the clinical specimen. As positive controls, genomic SARS‐CoV‐2 RNA and genomic SARS‐CoV RNA were used for the ORF1ab and the E‐Gene assay, respectively, adjusted to the Ct values of 28 and 32. PCR was conducted with the AgPath‐ID One‐Step RT‐PCR Reagents kit (Applied Biosystems) using a Bio‐Rad CFX96 or Bio‐Rad Opus real‐time PCR cycler.

### 4.3 Blood and Serum Sampling and PBMC Isolation

Whole blood was collected in lithium heparin tubes for peripheral blood mononuclear cells (PBMC) isolation and SSTII advance (all Vacutainer, BD) for serology. SSTII advance tubes were centrifuged for 10 min at 1000*g* prior to removing serum. Serum aliquots were frozen at –20°C until further use. PBMCs were isolated by gradient density centrifugation according to the manufacturer's instructions (Leucosep tubes, Greiner; Biocoll, Bio&SELL).

### 4.4 SARS‐CoV‐2 IgG S1 and N ELISA

Anti‐SARS‐CoV‐2 IgG ELISA specific for spike subunit 1 (S1) and nucleocapsid (N) were performed with a 1:100 serum dilution using the commercial kits (EUROIMMUN Medizinische Labordiagnostika AG) according to the manufacturer's instructions and measured at a Tecan infinite M plex reader with Magellan Pro V7.4 software. The test results were considered positive with uncertainty within an OD ratio (defined as absorbance difference between control and study sample) of 0.8–1.1, and positive above >1.1

### 4.5 Anti‐IgG Immunoblot

Multiplex anti‐IgG Immunoblot specific for the spike subunit 1 (S1) and spike subunit 2 (S2) was performed using the EUROLINE Anti‐SARS‐CoV‐2‐Profil (IgG) kit (EUROIMMUN Medizinische Labordiagnostika AG) according to the manufacturer's instructions. The data was analyzed using the EUROLine Scan Software. The lower cut‐off was set at 11 for certainly negative values. The range from 11 to 18 indicates the values classified as positive with uncertainty.

### 4.6 Epitope‐Specific Antibody ELISA

400 nM of biotinylated peptide S809‐826 (Biotin‐Ttds‐PSKPSKR*SFIEDLLFNKV*‐OH (Ttds linker = N‐(3‐{2‐[2‐(3‐amino‐propoxy)‐ethoxy]‐ethoxy}‐propyl)‐succinamic acid, JPT Peptide Technologies) or mutated peptides as indicated in the figure legends were immobilized on a 96‐well Streptavidin plate (Steffens Biotechnische Analysen GmbH) for 1 h at RT. After blocking (1 h, 30°C), serum samples were diluted 1:100 and incubated for 1 h at 30°C. HRP‐coupled, anti‐human‐IgG secondary antibody (Jackson Immunoresearch) was diluted 1:5000 (Jackson Immunoresearch) and added to the serum samples for 1 h at 30°C, then HRP substrate was added (TMB, Kem‐En‐Tec). The reaction was stopped by adding sulfuric acid, and absorption was measured at 450 nm using a FlexStation 3.

### 4.7 Ex Vivo T Cell Stimulation

Freshly isolated PBMC were cultivated at a concentration of 5 × 10^6^ PBMC/mL in AB‐medium containing RPMI 1640 medium (Gibco) supplemented with 10% heat‐inactivated AB serum (Pan Biotech), 100 U/mL of penicillin (Biochrom), and 0.1 mg/mL of streptomycin (Biochrom). Stimulations were conducted with PepMix overlapping peptide pools (15 aa length with 11 aa overlaps, JPT Peptide Technologies) covering the N‐terminal (S‐I) and C‐terminal (S‐II) part of the spike glycoprotein. Single peptide stimulations were conducted with the following peptides: iCope WT (N´‐SFIEDLLFNKVTLAD‐C´) or the mutated iCope peptides with the following aa substitutions: S816D, F817L, I181V, I818L, E819F, D820N, L821K, L822V, L822F, F823T, N824L, K825A, V826D, V826L, T827A (all JPT Peptide Technologies). All stimulations (peptide pools and single peptides) were performed at final concentrations of 1 µg/mL per peptide. For negative control, the stimulation peptide solvent DMSO diluted 1:1 in PBS was used at the same concentration as in peptide‐stimulated tubes. CEFX Ultra SuperStim pool (1 µg/mL per peptide) (JPT Peptide Technologies) was used as positive stimulation control. For optimized costimulation, purified anti‐CD28 (clone CD28.2, BD Biosciences) was added to each stimulation at a final concentration of 1 µg/mL. Incubation was performed at 37°C, 5% CO_2_ for 16 h in the presence of 10 µg/mL brefeldin A (Sigma‐Aldrich) during the last 14 h. CD4^+^ T cell activation was calculated as a stimulation index (stim. index) = % of CD40L^+^4‐1BB^+^ CD4^+^ T cells in the stimulation/% of CD40L^+^4‐1BB^+^ CD4^+^ T cells in the unstimulated control. The dotted lines indicate a stim.index of 1.5 (positive with uncertainty) and 3 (definite positive). All values below 0.1 were set to 0.1 for display.

### 4.8 Flow Cytometry

Stimulations were stopped by incubation in 2 mM EDTA for 5 min. Surface staining was performed for 15 min in the presence of 1 mg/mL of Beriglobin (CSL Behring) with the following fluorochrome‐conjugated antibodies titrated to their optimal concentrations: anti‐CD3‐FITC (Miltenyi Biotec), anti‐CD4‐VioGreen (Miltenyi Biotec), anti‐CD8‐VioBlue (Miltenyi Biotec), anti‐CD38‐APC (Miltenyi Biotec), and anti‐HLA‐DR‐PerCpVio700 (Miltenyi Biotec). During the last 10 min of incubation, Zombie Yellow fixable viability staining (Biolegend) was added. Fixation and permeabilization were performed with eBioscience FoxP3 fixation and PermBuffer (Invitrogen) according to the manufacturer's protocol. Intracellular staining was carried out for 30 min in the dark at room temperature with anti‐4‐1BB‐PE (Miltenyi Biotec), anti‐CD40L‐PEVio770 (Miltenyi Biotec), anti‐IFN‐γ (Biolegend), and anti‐TNF‐α V605 (Biolegend). All samples were measured on a MACSQuant Analyzer 16 (Miltenyi Biotec). Instrument performance was monitored prior to every measurement with Rainbow Calibration Particles (BD Biosciences). A representative gating strategy for the identification of antigen‐specific T cells and T cell avidity can be found in Figure S6.

### 4.9 HLA Typing and Analysis

HLA typing was performed by LABType CWD assays (One Lambda, West Hills, CA, USA) based on reverse sequence‐specific oligonucleotides (rSSO) according to the manufacturer's instructions. Briefly, the HLA genomic region was amplified individually using locus‐specific biotinylated primers for HLA‐DRB1, ‐DQA1, ‐DQB1, ‐DPA1, and –DPB1. Amplicons were hybridized to HLA allele‐ and allele‐group‐specific probes attached to Luminex beads. Complementary binding was detected by the addition of R‐phycoerythrin‐conjugated streptavidin and acquired using a FLEXMAP 3D flow analyzer (Luminex, Austin, TX, USA). HLA alleles were derived at two‐field code resolution (highest probability) as referenced in the catalog of common and well‐documented HLA alleles version 2.0.0 33. 12 individuals with a mean stimulation index across iCope/mutations outside 1.5 times the interquartile range from the upper or lower quartile were removed as outliers. MHCII peptide binding predictions were then generated for all combinations of the iCope/mutations peptides and alleles using the IEDB tools api (v2.26). All lengths from 11 to 15 were selected, and the percentile rank was taken as the binding rank. Results where the allele was only typed to the first field (e.g., DPA1*02) and alleles with unavailable prediction methods were discarded. Each individual's binding prediction results were collated into a list of all the results for their two DRB1 alleles, their four combinations of DQ, and their four combinations of DP. Each donor would then have 16 sets of collated binding results, one for each peptide. The median of the best 20 prediction ranks was calculated for each allele/peptide combination. Where 20 predictions were not available, all available predictions were used. For the MCHII‐medianbest20ranks/stimindex graphs, each individual/peptide combination is a separate datapoint.

### 4.10 Single‐Cell RNA Sequencing

For single‐cell RNA sequencing, PBMC of six donors with no history of SARS‐CoV‐2 infection and negative values for SARS‐CoV‐2 IgG were stimulated with 1 µg/mL iCope peptide in the presence of purified anti‐CD28 (clone CD28.2, BD Biosciences) and anti‐CD40 (clone HB14, Miltenyi Biotec). CD4^+^ T cells were enriched by MACS (Miltenyi Biotec) and CD40L^+^4‐1BB^+^ and CD40L^−^4‐1BB^−^ cells FACS sorted using an Aria SORP (BD). The cells were loaded with a maximum concentration of 1000 cells/µL and a maximum cell number of 17,000 cells on a Chromium Chip G (10x Genomics). Gene expression and TCR libraries were generated according to the manufacturer's instructions using the Chromium Next GEM single cell 5`Library and Gel bead Kit V1.1 and Chromium Single Cell V(D)J Enrichment Kit for human T cells (10x Genomics). Sequencing was conducted with a NovaSeq 6000 cartridge (Illumina) with 20,000 reads per cell for GEX libraries and 5000 reads per cell for TCR libraries.

### 4.11 Single‐Cell Transcriptome Analysis

Single‐cell RNA was mapped to the reference genome GRCh38‐2020‐A and preprocessed using the Cell Ranger Software v6.1.2 (10x Genomics). Quality control and analysis of data was done in R 4.0.5 (R Core Team (2021). R: A language and environment for statistical computing. R Foundation for Statistical Computing, Vienna, Austria. URL https://www.R‐project.org/) using the “Seurat” package [70]. To remove low‐quality cells, doublets, and empty cells, thresholds were set to 840–3000 RNA features and less than 5% mitochondrial RNA. Data were normalized by using the LogNormalize function of the Seurat package, and genes detected in less than 0.1% of the cells were excluded. For gene expression analysis, the TCR genes were excluded from the dataset to avoid TCR‐biased clustering. tSNE plots of CD40L^+^4‐1BB^+^ or CD40L^−^4‐1BB^−^ cells from young and older donors were overlaid using the in‐built plot function of the Seurat package. Following gene signatures were analyzed: T naïve/memory (SELL, LEF1, TCD7, CCR7, CD27, CD28), cytotoxicity (PRF1, GNLY, NKG7, GZMB), cytokines (IL‐2, IFN‐γ, TNF‐α, TGFβ1), senescence & exhaustion (LCK, ZAP70, LAT, TIGIT, CTLA4, BTLA, CD244, CD160, BATF, LCP2, TOX, PDCD1, KLRG1, HAVCR2 (TIM3), LAG3), and anergy (EGR2, EGR3 DGKα, GRAIL, CD200) [71, 72, 73, 74]. Normalized and log2‐transformed expression values of signature genes were shown in a heatmap using the DoHeatmap function of the Seurat package. Zero expression values were set to white color.

### 4.12 Singe Cell TCR Analysis

Single‐cell TCR data were preprocessed using the Cell Ranger Software v6.1.2 (10x Genomics) and the GRCh38‐2020‐A reference genome. Data were further processed in R using the “immunarch” package (https://CRAN.R‐project.org/package=immunarch, R package version 0.6.7.). Only cells that passed the quality controls in the gene expression analysis and contained exactly one TCR alpha and one TCR beta chain were used for further analysis. Diversity indices and rare clonal proportions were calculated using the corresponding functions of the immunarch package.

### 4.13 Data Analysis and Statistics

Sequence data for analysis of the mutational landscape of SARS‐CoV‐2 were from the GISAID EpiCov database (https://www.epicov.org/). Study data were collected and managed using REDCap electronic data capture tools hosted at Charité [75, 76]. Flow cytometry data were analyzed with FlowJo 10.6 (FlowJo LLC), and statistical analysis was conducted with GraphPad Prism 9. If not stated otherwise, data are plotted as mean. *N* indicates the number of donors. *p*‐values were set as follows: *p < 0.05, **p < 0.01, and ***p < 0.001.

## Author Contributions

Lucie Loyal: Conceptualization, investigation, data curation, visualization, formal analysis, and writing. Karsten Jürchott: Data curation, visualization, and formal analysis. Ulf Reimer: Data curation, visualization, and resources. Lil Meyer‐Arndt: Resources. Larissa Henze: Data curation and resources. Jak Kostrzanowski: Visualization and formal analysis. Bernhard Reus: Formal analysis. Norbert Mages, Maike Mangold, Beate Kruse, Manuela Dingeldey, Birgit Sawitzki, Janine Michel, Marica Grossegesse, Karsten Schnatbaum, Holger Wenschuh, Andreas Nitsche, Nils Lachmann, and Bernd Timmermann: Resources. Claudia Giesecke‐Thiel: Writing. Julian Braun: Data curation and resources. Florian Kern: Visualization, formal analysis, and resources. Andreas Thiel: Funding acquisition and writing.

## Funding

This work was funded by the Federal Ministry of Health through a resolution of the German Bundestag (Charité Corona Cross (CCC) 2.0 and 2.1 and Charité Corona Protect (CCP)). This publication was supported by the German Federal Ministry of Education and Research (BMBF) as part of the Network University Medicine (NUM): NaFoUniMedCovid19 grant no.: 01KX2021, COVIM.

## Ethics Statement

This study was approved by the Institutional Review Board of the Charité (EA/152/20).

## Patient Consent Statement

Written informed consent was obtained from all included participants, and the study was conducted in agreement with the Declaration of Helsinki.

## Conflicts of Interest

U.R. was and F.K. is an employee, and H.W. was the CEO of JPT. The usage of CD3 downregulation as a method for direct analysis of functional avidity of T cells was patented by L.L. and A.T. The remaining authors declare no conflicts of interest.

## Peer Review

The peer review history for this article is available at https://publons.com/publon/10.1002/eji.202551888.

## Supporting information




**Supporting File 1**: eji6015‐sup‐0001‐SuppMat.pdf

**Supporting File 1**: eji6015‐sup‐0002‐Tables.docx

## Data Availability

All flow cytometric data are accessible at https://doi.org/10.5281/zenodo.14938019 (CC‐BY license). Single‐cell RNA sequencing data have been deposited at the EGA (accession number EGAS50000001150) and will be made available upon completion of a data transfer agreement. Requests referencing this publication and the EGA identifier can be directed to datathiellab@charite.de.
