# Supplementary material for: Aging and Viral Evolution Impair Immunity Against Dominant Pan‐Coronavirus‐Reactive T Cell Epitope
Source: Eur J Immunol. 2025 Jul 28;55(7):e51888. doi: 10.1002/eji.202551888 (PMC12304627; doi:10.1002/eji.202551888)
Supplement: Supplementary file 1 — Supporting File 1: eji6015‐sup‐0001‐SuppMat.pdf [file EJI-55-e51888-s002.pdf]

Figure S1

A

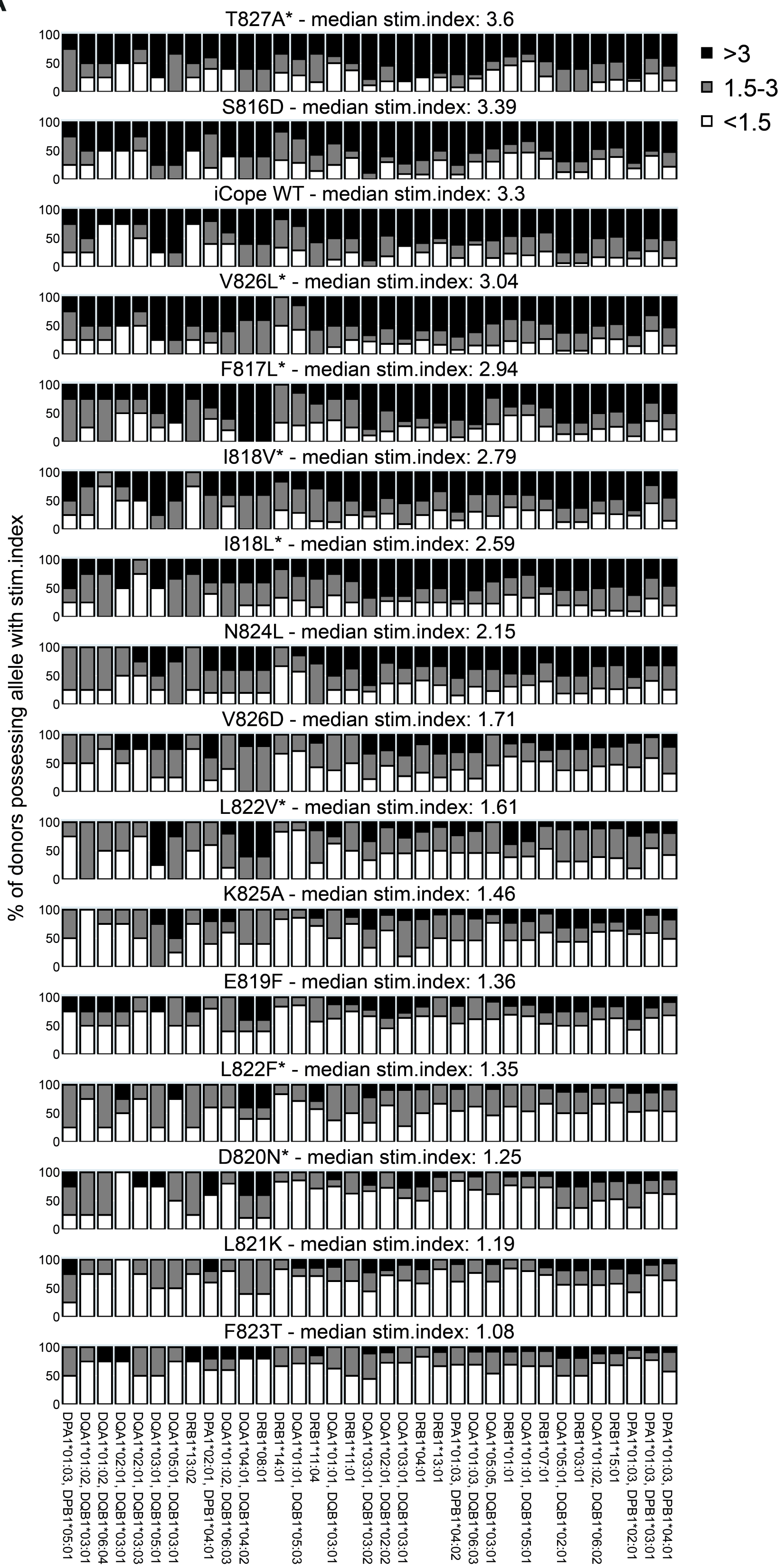
T827A* - median stim.index: 3.6
S816D - median stim.index: 3.39
iCope WT - median stim.index: 3.3
V826L* - median stim.index: 3.04
F817L* - median stim.index: 2.94
I818V* - median stim.index: 2.79
I818L* - median stim.index: 2.59
N824L - median stim.index: 2.15
V826D - median stim.index: 1.71
L822V* - median stim.index: 1.61
K825A - median stim.index: 1.46
E819F - median stim.index: 1.36
L822F* - median stim.index: 1.35
D820N* - median stim.index: 1.25
L821K - median stim.index: 1.19
F823T - median stim.index: 1.08
% of donors possessing allele with stim.index
>3
1.5-3
<1.5
DPA1*01:03, DPB1*05:01
DQA1*01:02, DQB1*03:01
DQA1*01:02, DQB1*06:04
DQA1*02:01, DQB1*03:01
DQA1*02:01, DQB1*03:03
DQA1*03:01, DQB1*05:01
DQA1*05:01, DQB1*03:01
DRB1*13:02
DPA1*02:01, DPB1*04:01
DQA1*01:02, DQB1*06:03
DQA1*04:01, DQB1*04:02
DRB1*08:01
DRB1*14:01
DQA1*01:01, DQB1*05:03
DRB1*11:04
DQA1*01:01, DQB1*03:01
DRB1*11:01
DQA1*03:01, DQB1*03:02
DQA1*02:01, DQB1*02:02
DQA1*03:01, DQB1*03:01
DRB1*04:01
DRB1*13:01
DPA1*01:03, DPB1*04:02
DQA1*01:03, DQB1*06:03
DQA1*05:05, DQB1*03:01
DRB1*01:01
DQA1*01:01, DQB1*05:01
DRB1*07:01
DQA1*05:01, DQB1*02:01
DRB1*03:01
DQA1*01:02, DQB1*06:02
DRB1*15:01
DPA1*01:03, DPB1*02:01
DPA1*01:03, DPB1*03:01
DPA1*01:03, DPB1*04:01

Fig. S1: Impact of different alleles on T cell responsiveness against iCope mutations. Lines indicate the percentage of different stim responses (y-axis) for all donors who possess the given allele (x-axis). Alleles with frequencies > 5% in the analyzed cohort (all donors of Table S1) are shown. Each donor is represented with at least one allele combination. The color code indicates the percentage of donors responding to the T cell stimulation with the respective peptide with a stimulation index < 1.5 (white, unresponsive), 1.5-3 (grey, weak response) or > 3 (black, strong response). * indicates documented iCope mutations.

Figure S2

A

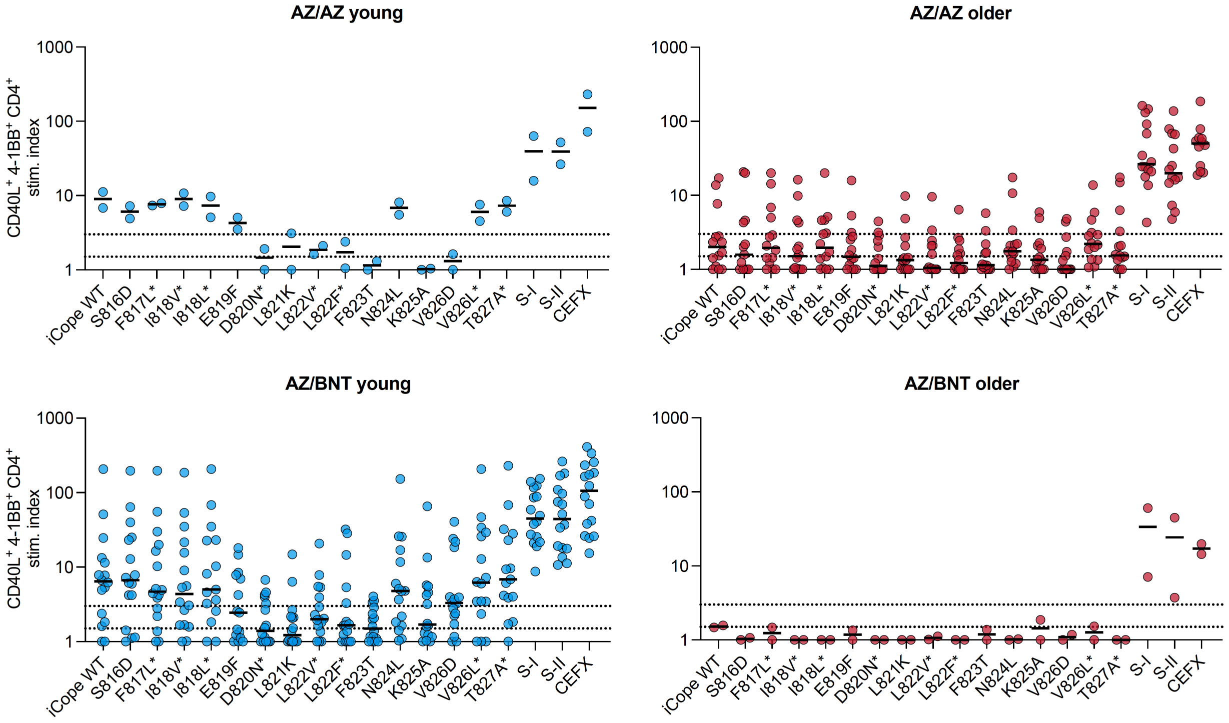

Fig. S2: iCope responsiveness is age dependent but independent of the vaccine scheme. Ex vivo stimulation of PBMCs from young or older AZ/AZ (*n*=2/14), and AZ/BNT (*n*=16/2) vaccinated individuals with iCope WT or different mutated iCope peptides and control pools S-I, S-II and CEFX. Bars show the median. Dotted lines indicate a stim. index of 1.5 and 3. All values below 0.1 were set at 0.1 for display. * indicates documented iCope mutations. AZ: ChAdOx1 COVID-19 vaccine (AstraZeneca).

Figure S3

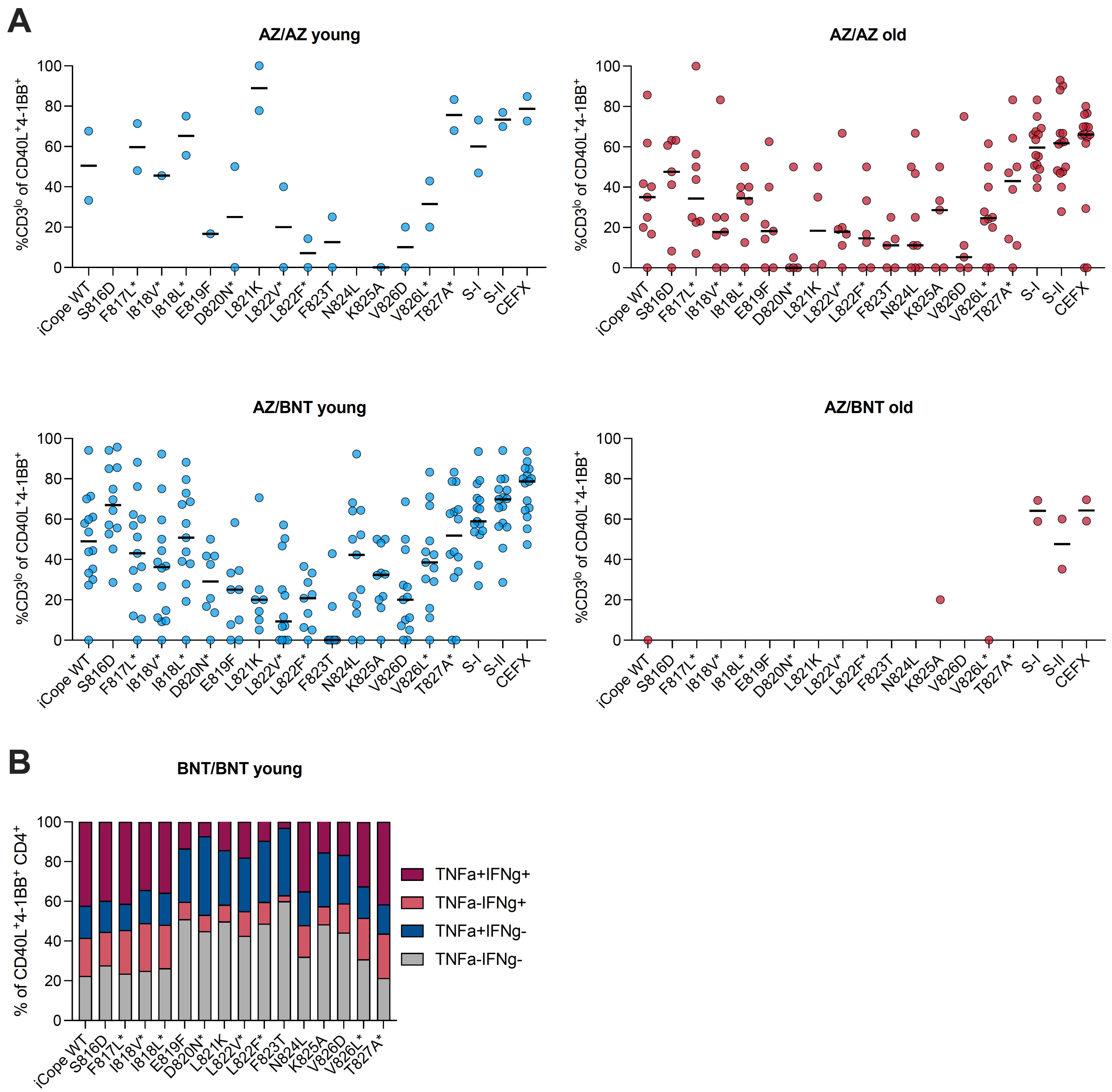

Fig. S3: CD3^{lo} T cells in AZ/AZ and AZ/BNT vaccinated and mutations affecting the cytokine profile. (A) Ex vivo stimulation of PBMCs from young and older AZ/AZ (*n*=2/14) and AZ/BNT vaccinated (*n*=16/2) individuals with iCope WT or different mutated iCope peptides and the control pools S-I, S-II and CEFX. Frequencies of CD3^{lo} cells in CD40L^{+}4-1BB^{+} CD4^{+} T cells are shown for T cell responses with a stim. index $\geq$1.5. Bars show the median. **(B)** Proportion of IFN-γ and/or TNF-α producing T cells among CD40L^{+}4-1BB^{+} CD4^{+} T cells in the BNT/BNT young after stimulation with iCope WT or the different mutated peptides. * indicates documented iCope mutations.

Figure S4

A

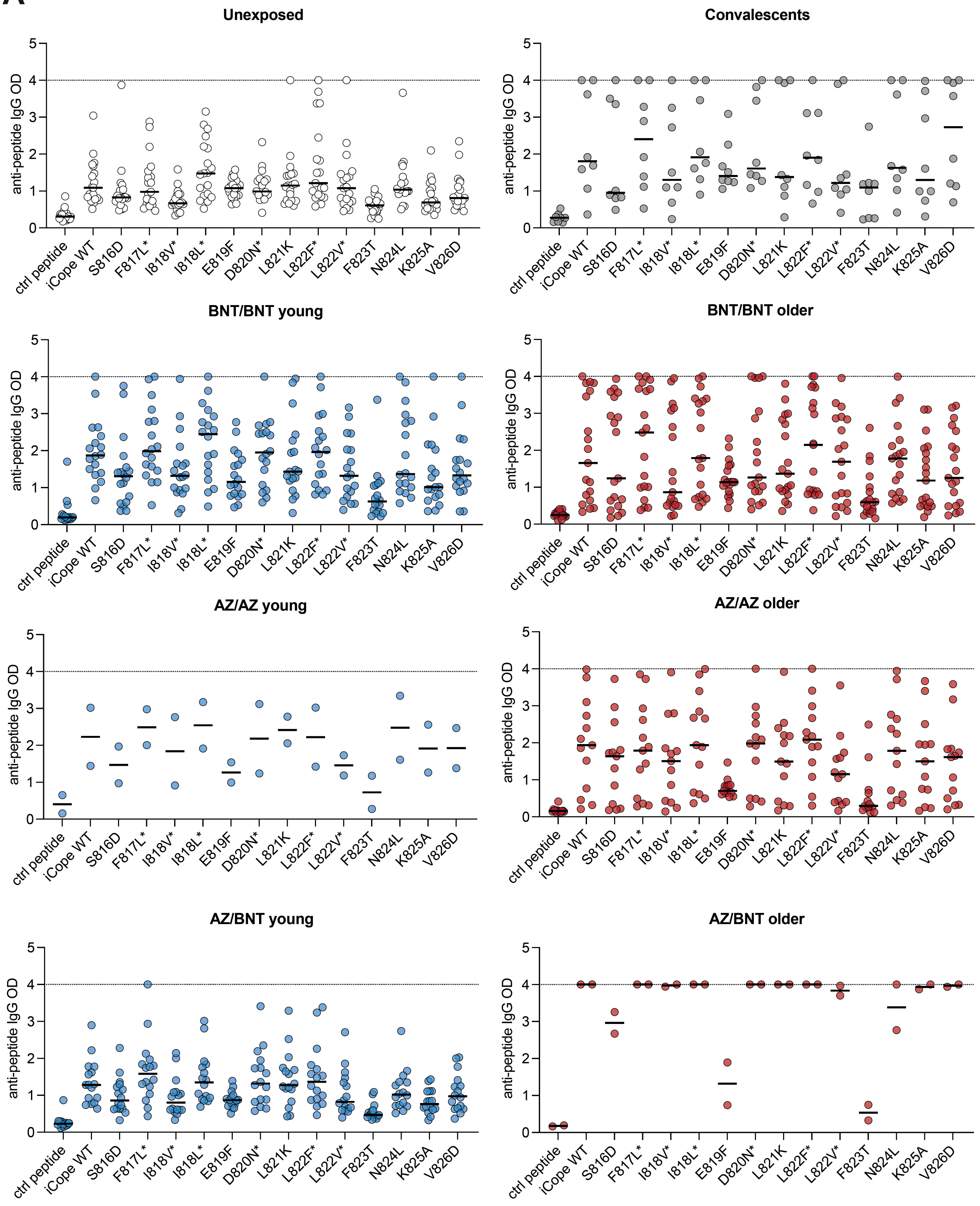

Fig. S4: Anti-S809-826 humoral immune responses are affected by mutations but not by age.
(A) Optical density of anti-S809-826 peptide IgG (ELISA) relative to indicated mutations. Peptide

S1133-1147 was used as internal control (ctrl peptide). Bars show the median. Dotted line indicates the upper detection limit. * indicates documented iCope mutations.

Figure S5

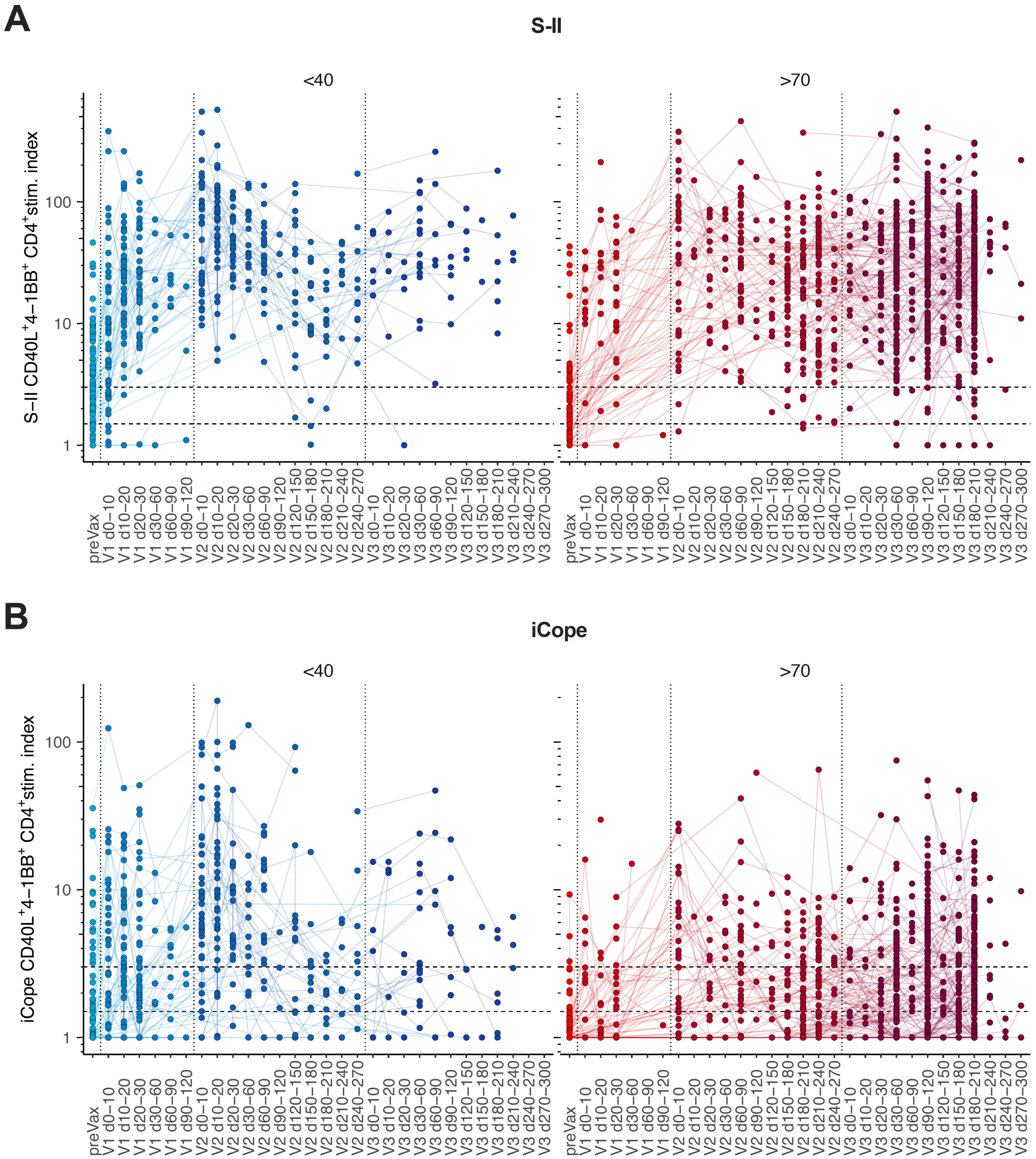

Fig. S5: S-II and iCope responsiveness over time in young and older. (A) Ex vivo stimulation of PBMCs from young (<40 years, blue) or older (>70 years, red) individuals with S-II prior to first vaccination (preVax) or at indicated timespans after the first, second or third dose of vaccine (V1-3). **(A, B)** Individual resolution of SI after stimulation with S-II **(A)** or iCope **(B)**. Repeated measurements of the same individuals are connected with lines.

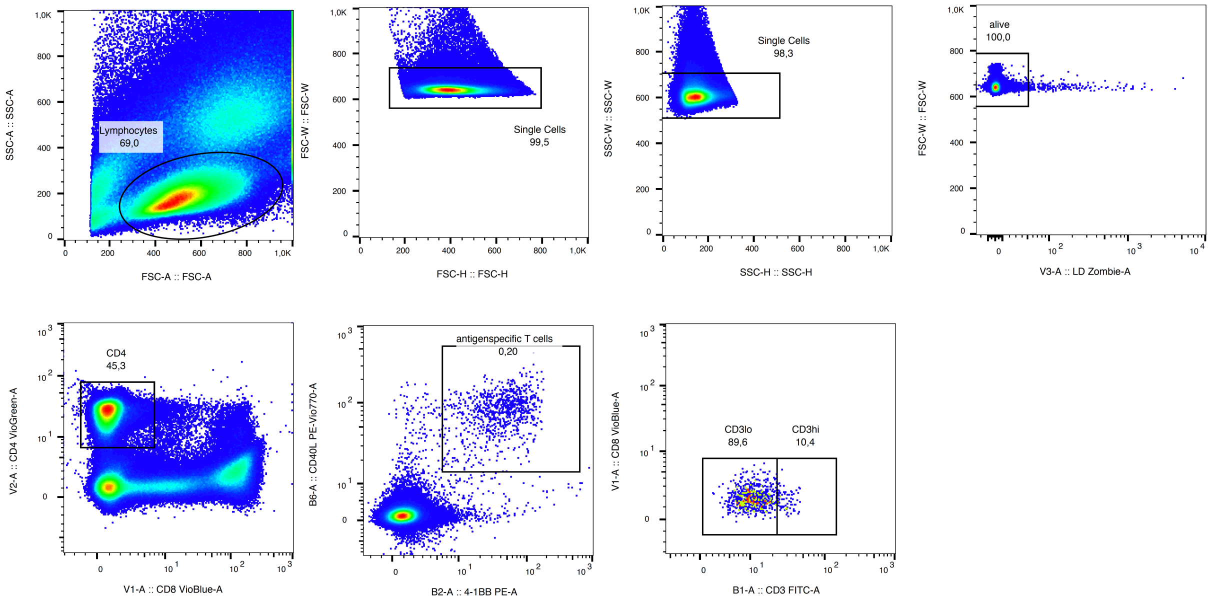

Fig. S6: Exemplary gating strategy.
