# Supplementary material for: Aging and Viral Evolution Impair Immunity Against Dominant Pan‐Coronavirus‐Reactive T Cell Epitope
Source: Eur J Immunol. 2025 Jul 28;55(7):e51888. doi: 10.1002/eji.202551888 (PMC12304627; doi:10.1002/eji.202551888)
Supplement: Supplementary file 2 — Supporting File 1: eji6015‐sup‐0002‐Tables.docx [file EJI-55-e51888-s001.docx]

**Supporting Information**

**Tables:**

| Cohort | Donor number  (#) | Gender  (# females) | Mean age and [SD] (years) | Mean time since infection/ 2nd dose [SD] (days) |
| --- | --- | --- | --- | --- |
| Unexposed | 17 | 12 | 26.7 [4.2] | - |
| Convalescents | 8 | 7 | 43.6 [12.2] | 286 [57] |
| BNT/BNT young | 18 | 11 | 30.0 [7.1] | 24 [6.7] |
| BNT/BNT older | 19 | 7 | 76.7 [3.9] | 22 [3.1] |
| AZ/BNT young | 16 | 9 | 27.0 [5.8] | 83 [3.6] |
| AZ/BNT older | 2 | 1 | 63.0 [4.2] | 75 [12.7] |
| AZ/AZ young | 2 | 2 | 30.5 [4.9] | 78 [0.7] |
| AZ/AZ older | 14 | 7 | 71.4 [3.4] | 78 [5.9] |

**Table S1: Donor characteristics T cell stimulations and serology.**

| Cohort | Donor number  (#) | Gender  (#females) | Mean age and [SD] (years) | Time since 3rd dose/infection  [SD] (days) |
| --- | --- | --- | --- | --- |
| Young vaccinated | 1-3 | 2 | 29.3 [1.5] | 85.7 [8.1] |
| Young vaccinated and infected | 4-9 | 6 | 31.3 [7.3] | 46.1 [33.7] |
| Older vaccinated | 10-12 | 2 | 82.3 [1.5] | 80 [12.2] |
| Older vaccinated and infected | 13-17 | 1 | 81.0 [3.4] | 54.2 [35.6] |

**Table S2: Donor characteristics single-cell RNA-sequencing.**

| Cohort | allergy | cardio | lung | rheuma | gastroin | diabetes | tumor |
| --- | --- | --- | --- | --- | --- | --- | --- |
| Unexposed | 5 | 1 | 0 | 0 | 0 | 0 | 0 |
| Convalescents | 4 | 3 | 1 | 1 | 0 | 0 | 1 |
| BNT/BNT y | 9 | 1 | 1 | 1 | 1 | 0 | 1 |
| BNT/BNT o | 8 | 11 | 3 | 0 | 1 | 1 | 5 |
| AZ/BNT y | 3 | 0 | 0 | 0 | 0 | 0 | 0 |
| AZ/BNT o | 1 | 1 | 0 | 0 | 1 | 0 | 0 |
| AZ/AZ y | 1 | 0 | 0 | 0 | 2 | 0 | 0 |
| AZ/AZ o | 8 | 9 | 2 | 1 | 2 | 0 | 3 |
| scRNA vac y | 1 | 0 | 0 | 0 | 0 | 0 | 0 |
| scRNA vac/inf y | 0 | 0 | 0 | 0 | 0 | 0 | 0 |
| scRNA vac o | 1 | 2 | 0 | 0 | 0 | 0 | 1 |
| scRNA vac/inf o | 1 | 5 | 2 | 0 | 0 | 0 | 0 |

**Table S3: Overview comorbidities in analyzed cohorts.** y = young, o = old, vac = vaccinated, inf = infected, cardio = cardiovascular disease, lung = lung diseases, rheuma = rheumatic diseases, gatstroin = disease of the gastrointestinal tract, tumor = history of cured tumor(s).
